# Vaccines against candidiasis: Status, challenges and emerging opportunity

**DOI:** 10.3389/fcimb.2022.1002406

**Published:** 2022-08-18

**Authors:** Satya Ranjan Sahu, Swagata Bose, Manish Singh, Premlata Kumari, Abinash Dutta, Bhabasha Gyanadeep Utkalaja, Shraddheya Kumar Patel, Narottam Acharya

**Affiliations:** ^1^ Laboratory of Genomic Instability and Diseases, Department of Infectious Disease Biology, Institute of Life Sciences, Bhubaneswar, India; ^2^ Regional center of Biotechnology, Faridabad, India; ^3^ School of Biotechnology, Kalinga Institute of Industrial Technology, Bhubaneswar, India

**Keywords:** Mycosis, *Candida*, drug resistance, immunity, whole cell vaccine, pan-fungal vaccine, antibody

## Abstract

Candidiasis is a mycosis caused by opportunistic *Candida* species. The occurrence of fungal infections has considerably increased in the last few years primarily due to an increase in the number of immune-suppressed individuals. Alarming bloodstream infections due to *Candida* sp. are associated with a higher rate of morbidity and mortality, and are emerged as major healthcare concerns worldwide. Currently, chemotherapy is the sole available option for combating fungal diseases. Moreover, the emergence of resistance to these limited available anti-fungal drugs has further accentuated the concern and highlighted the need for early detection of fungal infections, identification of novel antifungal drug targets, and development of effective therapeutics and prophylactics. Thus, there is an increasing interest in developing safe and potent immune-based therapeutics to tackle fungal diseases. In this context, vaccine design and its development have a priority. Nonetheless, despite significant advances in immune and vaccine biology over time, a viable commercialized vaccine remains awaited against fungal infections. In this minireview, we enumerate various concerted efforts made till date towards the development of anti-*Candida* vaccines, an option with pan-fugal vaccine, vaccines in the clinical trial, challenges, and future opportunities.

## Introduction

In addition to viruses and bacteria, fungi are the other organisms, that are either equally or more dangerous to human health. Out of about 4 million diverse fungal species, ~300 species have been identified as human pathogens to cause diseases (Hawksworth and Lucking, 2017). Candidiasis, aspergillosis, mucormycosis, and cryptococcosis are standout fungal infections. Despite the currently available diagnosis and treatment, approximately 1.5 million infectants succumb per year accounting for fungal infections worldwide, and the fatality rate is very similar to that caused by *Mycobacterium tuberculosis* (Mtb) or human immunodeficiency virus (HIV) and is more than the deaths due to malaria or breast or prostate cancer (Brown et al., 2012). Among these fatalities, *Candida* species alone are primarily accountable for the bulk of fungal infections. Fungal infections caused by *Candida* species range from superficial mucosal candidiasis such as vulvovaginal candidiasis (VVC) and oropharyngeal candidiasis to life threatening bloodstream infections such as candidemia. Systemic candidiasis is frequently reported as a consequence of intestinal dysbiosis, impaired host immunity, and high-risk associated medical therapy like immunosuppressive therapy, central venous catheters, or surgical interventions (Butler et al., 2009; Garcia-Vidal et al., 2013; Manohar et al., 2018; Peroumal et al., 2019). Candidiasis represents the 4th and 6th most common healthcare-associated bloodstream infections in the United States and Europe, respectively (Marchetti et al., 2004; Kim and Sudbery, 2011). CDC estimates ~10 per 100,000 people of candidemia incidences and nearly 25,000 such cases nationwide each year. Candidemia is estimated to affect nearly half a million patients per year worldwide (Tso et al., 2018). Since candidemia is usually diagnosed using blood cultures, about 50% of invasive *Candida* infections are undetected as the infections hit the essential internal organs like the heart, kidney, bones, etc. (Berenguer et al., 1993). Therefore, the existing epidemiology and incidence of invasive candidiasis date is most likely imprecise and underestimated, and it could be nearly 2-3 folds higher than that reported. The lack of a rapid diagnosis using a reliable and accurate methodology is yet another concern to *Candida* management. The late and poor diagnosis of invasive candidiasis has only contributed to the rise in multi-organ failures by septicemia-associated fatality. *Candida albicans* is by far the major cause of infections among all *Candida* species, trailed by *Candida glabrata, Candida parapsilosis, Candida tropicalis*, and *Candida krusei*. Moreover, the recent emergence of drug resistant *Candida auris* has challenged the existing healthcare system (Allert et al., 2022).

Much of the available information and strategy for overcoming bacterial and viral infections have also been deployed for curbing the fungal invasion. As of today, chemotherapy is the sole available option for overcoming fungal diseases. Polyenes, echinocandins, and azoles are the three major classes of antifungals currently being prescribed (Ford et al., 2015; Lee et al., 2021). These antifungals exhibit a narrow spectrum of activity to work only against certain fungal species and cytotoxicity, and often cause side effects. Azoles are the widely prescribed drugs that target ergosterol biosynthesis pathways. They inhibit the accumulation of ergosterol in the fungal cell membrane and fluidity. Although not many reports are available to suggest immediate side effects of the use of azoles, liver toxicity, and hormone-related adverse effects have been associated with the prolonged use of azoles (Benitez and Carver, 2019). Amphotericin B of the polyene group also acts on ergosterol to alter fungal cell membrane permeability, but it is nephrotoxic and expensive. Unlike azoles and amphotericin B, the echinocandin class of drugs inhibits 1,3-β glucan synthase by noncompetitive inhibition and reduces β-glucan deposition on the fungal cell wall. Caspofungin was the first intravenously administered antifungal in this group. The caspofungin users suffer from fever, headache, allergic reactions, and local inflammation of the veins (Kounis, 2013). Despite the advances accomplished so far, overcoming and eliminating the fungal infection has remained a domain of concern for healthcare professionals, globally. In addition to these limited options of anti-fungal drugs, continuous rise in the number of cases, the emergence of drug resistance isolates, infections by varieties of fungal strains, increase in immuno-compromised hosts, etc. further advocate for the urgency to develop better diagnostics, novel antifungal drugs, immunotherapeutics, and fungal vaccines.

In this review, we have emphasized the fight against *Candida* infections, more importantly, the current status of developing a successful vaccine. Vaccines against invasive pathogens represent a major step forward in combating illnesses, and developing an effective and successful anti-*Candida* vaccine is an apparent way to prevent candidemia. In recent years, based on the studies of host-fungal interaction, several groups have reported the immunogenicity and efficacy of different kinds of potential vaccine candidates against *Candida* in animal models (Ibrahim et al., 2006; Spellberg et al., 2006; Spellberg et al., 2006). Even few potential vaccines have been found to be effective and safe in initial clinical trials (Schmidt et al., 2012; De Bernardis et al., 2012). Through this review, we enumerate the multiple concerted initiatives made to date in the formulation of several categories of *Candida* vaccines, an option with pan-fungal vaccine, vaccines in a clinical trial, and challenges in developing a successful anti- candidiasis vaccine ahead with us.

## *Candida* vaccines

**Figure 1:**
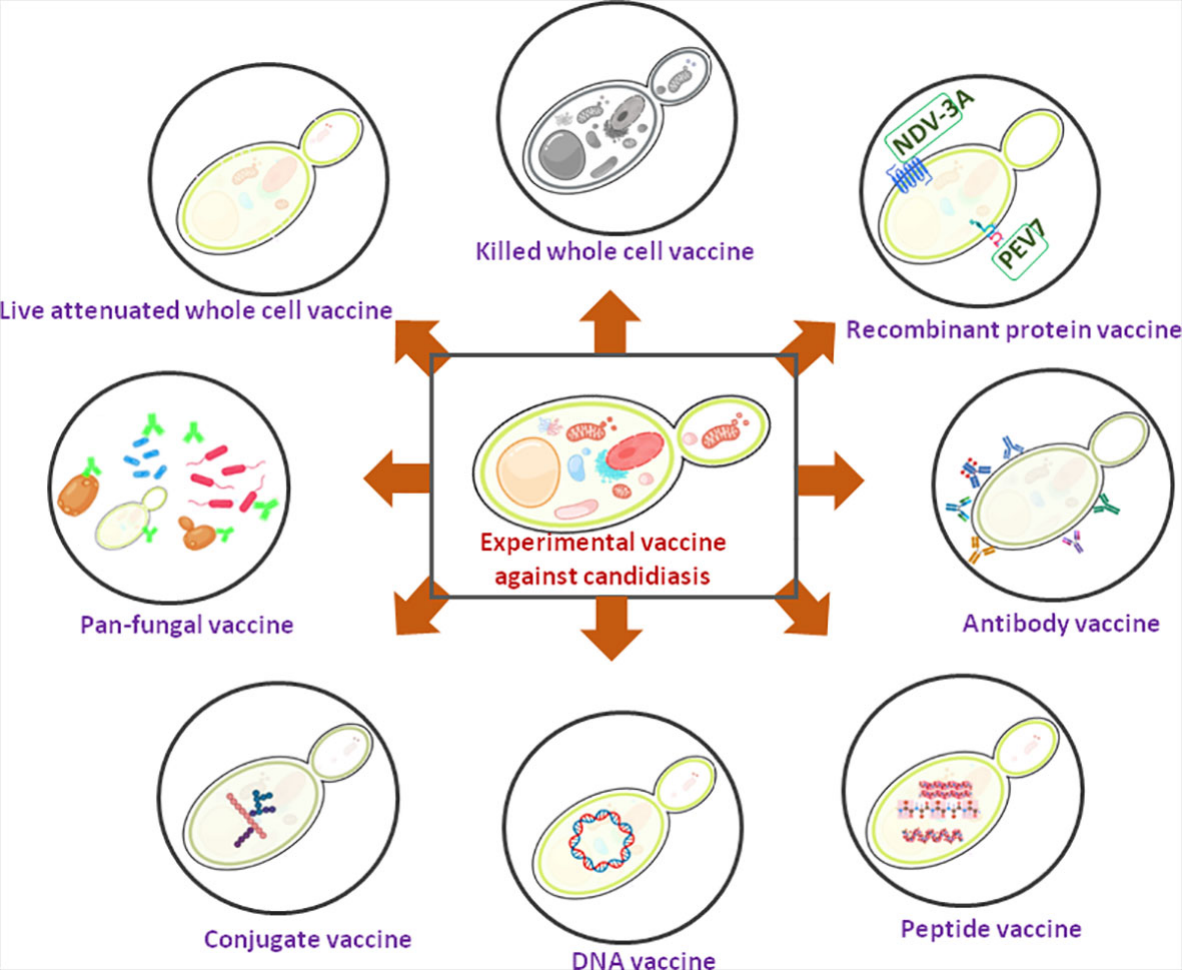
A schematic diagram showing experimental *Candida* vaccines. Two recombinant protein vaccines are under clinical trial (showing green).

Vaccines play a critical role in preventing deaths, hospitalization, and the spreading of diseases caused by infectious agents. As a protective and preventive strategy, various vaccination programs have been placed in several countries and achieved remarkable success in reducing morbidity and mortality associated with various infections. Vaccinations prevent 6 million fatalities per year all across the world (Andre et al., 2008). Even after prolonged efforts, currently, there is no commercialized anti-*Candida* vaccine that has been approved for human use (Santos and Levitz, 2014). However, several anti-*Candida* experimental vaccines have been identified during the last few decades, and two of them have been undertaken for clinical trials (Tso et al., 2018). Since the fatality rate due to fungal infections by the drug resistant strains and the number of immunocompromised individuals are on the rise, the development of an effective fungal vaccine and its successful implementation will be a great help to mankind. An updated summary of available potential *Candida* vaccine candidates has been given below (**Figure 1** and **Table 1**).

**Table 1:** Summary of experimental anti-*Candida* vaccine candidates.

Vaccine category	Description	Clinical trial stage	Cross-protection (non-*C. albicans* organisms)	Reference
LA	Hyphal-defective *C. albicans* strain PCA-2		*S. aureus*	Bistoni et al. (1986)
LA	MAP kinase-defective *hog1*Δ *C. albicans* strain CNC13			Fernández‐Arenas et al. (2004)
LA	Cell wall-defective *ecm33*Δ *C. albicans* strain RML2U			Martínez‐López et al. (2008)
LA	Filamentation-repressible *C. albicans* strain tet-NRG1			Saville et al. (2009)
LA	Yeast-locked *C. albicans* strain *cph1*Δ/*efg1*Δ			Yang et al. (2009)
KV	heat-killed *S. cerevisiae* yeast (HKY)		*A. fumigatus, C. grubii*, and *C. posadasii*	(Liu et al. 2012), Liu et al. (2011a), Capilla et al. (2009)
Conjugate	laminarin-CRM conjugation			Torosantucci et al. (2005)
Conjugate	β-glucan coupled with a MF59			Pietrella et al. (2010)
Conjugate	Lam-CRM197, a laminarin conjugated with diphtheria toxoid (CRM197)			Cassone et al. (2010)
Conjugate	Lam-CRM197 coupled with MF59			Wang et al. (2015)
Recombinant	recombinant N-terminus of Als1 (rAls1p-N)			Ibrahim et al. (2006)
Recombinant	recombinant N-terminus of Als3 (rAls3p-N)		*S. aureus*	Spellberg et al. (2006)
Recombinant	Recombinant N-terminus of Als3p plus alum adjuvant (NDV-3)	Phase I b/2a clinical trial		Schmidt et al. (2012)
Recombinant	recombinant Sap2p			De Bernardis et al. (2012)
Recombinant	PEV7, a truncated Sap2p embedded into the bilipid layer of an influenza virosomes	Phase I clinical trial		De Bernardis et al. (2012)
Recombinant	47-kDa fragment of *C. albicans* Hsp90’s from its carboxyl end (Mycograb)			Matthews et al. (1987), Matthews et al. (1989)
Recombinant	recombinant form of N-terminus of Hyr1 (rHyr1p-N)		*C. glabrata, C. krusei, C. parapsilosis* and *C. tropicalis*	Luo et al. (2011)
Recombinant	recombinant enolase (Eno1p)			Shibasaki et al. (2014)
Peptide	Fba (derived from *C. albicans* cell surface fructose bisphosphatealdolase [Fab]) or peptide Met6 (derived from *C. albicans* β-1,2–mannotriose [β-(Man)3] protein			Xin and Cutler (2006), Xin et al. (2008), Han et al. (1998), Xin et al. (2006)
Peptide	Dendritic cells (DCs) stimulated by either Fba peptide (YGKDVKDLFDYAQE) or Met6 peptide (PRIGGQRELKKITE)			Xin et al. (2016)
Peptide	Fba peptide (14-mer) conjugated with each one of five peptide mimotopes from Met6 (PS2, PS31, PS28, PS55, and PS76)			Xin (2019)
extracellular vesicles	fungal extracellular vesicles (EV)			Edwards et al. (2018)
Cell wall extract	β-mercaptoethanol-extracted *C. albicans* Cell wall proteins			Chaffin et al. (1998), Martínez et al. (1998)

LA, live attenuated; KV, Killed vaccine.

### Live-attenuated whole cell vaccines

Live attenuated vaccine is nothing but the whole pathogen that has just been “weakened” (attenuated) such that it elicits enough protective immune response but does not cause disease in healthy people. It causes a very mild infection that ensures a long-lasting protective immune memory. The concept of live attenuated vaccines came from the Vaccinia virus, which causes cowpox in cattle but cross-protects smallpox in humans (Riedel, 2005). Live-attenuated vaccine strategy is commonly and effectively used in combating viral diseases such as influenza, polio, mumps, rubella, measles, varicella, and rotavirus (Saccente and Woods, 2010; Leibovitch and Jacobson, 2016). Such approaches have been taken to develop a vaccine against SARS-COV2 also, and it appears to be the lifesaver against COVID19 (Sapkal et al., 2021). On the same line, several studies reported the ability of various avirulent or low-virulent strains of *C. albicans* to protect against candidiasis. *C. albicans* strain PCA-2 is a caspofungin resistant mutant of its isogenic 3153A strain. Mice immunized with PCA-2 elicit an innate immune response by increasing the number of peripheral blood polymorphonuclear cells (PMNs) with high candidacidal activity. Consequently, an adoptive transfer of macrophage cells from PCA-2 administered mice conferred substantial protection against subsequent infection (Bistoni et al., 1986). Fernandez-Arenas, E. et al., in their study used low virulent *C. albicans* strains CM1613 (a Mitogen Activated Protein Kinase MKC1 deletant), CNC13 (a MAP-kinase HOG1 deletant), and a morphologically defective mutant 92’ as whole cell vaccines in murine models (Fernandez-Arenas et al., 2004). Among these strains, only CNC13 strain immunized mice got partially protected (~60–70%) when they were re-challenged with a lethal dose of wild-type infection. This study further demonstrated that both the cellular and humoral responses were induced upon CNC13 immunization that elicited effective protection. This study also showed a clear difference in the antibody pattern in the sera of non-vaccinated and vaccinated animals with the CNC13 mutant. The cell wall acts as an interface between the host and fungal pathogens and its composition plays an important role in eliciting the host immune response. Ecm33, a glycosylphosphatidylinositol-anchored protein, is involved in the biogenesis and fungal cell wall integrity. The *C. albicans* strain RML2U (*ecm33Δ* Δ) is defective in its interaction with endothelial and epithelial cells because of its cell wall structure modification and was shown to protect vaccinated BALB/c mice (Martinez-Lopez et al., 2008). Nrg1 is a transcription factor that negatively regulates morphological transition in *C. albicans*. SSY50-B is a genetically modified *C. albicans* strain where the expression of *NRG1* is governed by a tetracycline-regulatable promoter. The absence or supplementation of doxycycline (DOX) to the growth media regulates the expression of *NRG1*, thereby, the morphology and virulence get manipulated. B cell deficient mice immunized with tet-NRG1mutat strain remained protected upon subsequent infection similar to wild type mice strain, while the same was not witnessed with T cell deficient mice. However, survival was only evidenced when tet-NRG1 mutant was enforced to grow in yeast form (Saville et al., 2009). Similarly, the double-deletion mutant *cph1ΔΔ/efg1*ΔΔ *C. albicans* is avirulent as it is locked in the yeast form (Lo et al., 1997). Although the *cph1ΔΔ/efg1*ΔΔ mutant cells proliferate in infected mice, its immunization only partially protects mice from systemic infections upon the lethal dose of virulent challenge. Even its booster dose did not improve the degree of protection (Yang et al., 2009). The *gpi7 C. albicans* mutant is yet another avirulent strain and its immunization protected mice against pan-fungal invasive candidiasis (Shen et al., 2020). In this strain, the β- (Hawksworth and Lucking, 2017; Peroumal et al., 2019)-glucans layer of the cell wall is exposed that facilitating Dectin-1 receptor dependent nuclear translocation of RelB in macrophages, the release of Interleukin 18, and the production of protective antibodies. The study also found that IgG antibodies present in the patients recovering from invasive candidiasis and gpi7 mutant-immunized mice are very similar. BCG (Bacillus Calmette-Guérin) is an antibacterial live attenuated vaccine that induces non-specific cross-protection against a wide range of infections (Mangtani et al., 2014). Immunization with BCG induces a protective innate immune response against pathogens like *C. albicans* and *Staphylococcus aureus* in addition to *Mycobacterium tuberculosis*, and this study provided a concept of “trained immunity”. Trained immunity provides an enhanced protective response to a secondary infection caused either by the same or different pathogens, mostly mediated by the innate immune system (Netea et al., 2011). Monocytes/macrophages and natural killer (NK) cells play critical roles in contributing to trained immunity, and such immunity is independent of T and B cell responses (Kleinnijenhuis et al., 2014). It was found that BCG vaccination induces epigenetic modifications by altering the methylation status of histones in the regulatory elements of associated genes in circulating monocytes that facilitate increased production of proinflammatory cytokines leading to a “trained” state (van 't Wout et al., 1992; Kleinnijenhuis et al., 2012). Despite these advances, most of the avirulent strains failed to reach a clinical trial stage. Possible reasons include (i) due to weakened immune systems, currently, it is not advocated to use in immunocompromised individuals, (ii) stability of the attenuation of the genetically modified or mutant strains is questionable as it was found in the case of viruses that the virulence is not suppressed permanently and they regained virulence in immune-deficient individual and caused infection (Lancaster and Pfeiffer, 2011), although similar reports do not exist yet to support in the case of attenuated fungal strains, (iii) though the vaccine candidates govern substantial and marked protection in animal models, some of them failed to show similar response in human volunteers at a clinical trial, and (iv) live attenuated vaccines share a common flaw with biologics in that they are difficult to keep stable during the manufacturing, transporting, and preservation phases (van 't Wout et al., 1992).

### Killed whole cell vaccines

In comparison to the live attenuated vaccines, killed vaccines are stable and non-pathogenic as they cannot revert. The ease and low cost of its preparation along with substantial safety make it a choice against the *Candida* vaccine. Intranasal vaccination with heat-killed *C. albicans* plus a heat-labile genetically engineered toxin from *Escherichia coli* as an adjuvant R192G provided a substantial degree of protection in animal models (Cardenas-Freytag et al., 1999). However, the same combination used for mucosal vaccination did not ensure full protection against experimental vaginal candidiasis (Cardenas-Freytag et al., 2002). Besides, this vaccine candidate has not been tested in immune-compromised animal models. The subcutaneous immunization with heat-killed *S. cerevisiae* yeast (HKY) also provided cross-protection to *C. albicans*, *A. fumigatus*, and *Coccidioides posadasii* infections (Capilla et al., 2009; Liu et al., 2011a; Liu et al., 2011b; Stevens et al., 2011). However, homologous proteins to components of HKY responsible for such cross-protection are yet to be demonstrated. Interestingly, the total cellular proteins extracted by β-mercaptoethanol treatment and their subsequent subcutaneous immunization to mice increased survivability (75%) with a decreased fungal burden than in the control groups (Thomas et al., 2006). A combined vaccine formulation of MV140 and V132 has been tested to prevent both bacterial as well as fungal genitourinary tract infections (GUTIs) (Martin-Cruz et al., 2020). The heat-inactivated polyvalent bacterial vaccine MV140 prevents RUTIs by eliciting Th1/Th17 and IL-10 immune responses, while the V132 is a heat-inactivated vaccine candidate developed against RVVCs. The report suggested that the vaccine combination activates human dendritic cells (DCs) to polarize potent IFN-γ and IL-17A double positive T cells and FOXP3^+^ regulatory T_reg_ cells. In addition, MV140/V132 promotes epigenetic reprogramming in human DCs to induce trained immunity. Even after these positive results in basic research, these candidates have not progressed to clinical trials.

### Conjugate vaccines

A conjugate vaccination combines a weak antigen with a robust antigen as a carrier, causing the immune system to respond more strongly to the weaker antigen. Conjugating proteins to polysaccharides facilitates easy recognition of the abundant fungal cell wall glycan components by the immune system, and thereby antibodies recognize the pathogen quickly. The first conjugate vaccine was developed against *Cryptococcus neoformans* where the capsular polysaccharide, glucuronoxylomannan (GXM) of the fungus was covalently linked to tetanus toxoid (TT). Vaccination of the conjugates with an adjuvant monophosphoryl lipid A (MPL) protected 70% of intravenously challenged mice (Devi, 1996). Similarly, a universal, pan-fungal vaccine was made by conjugating laminarin, a β-glucan polysaccharide isolated from brown algae, with inactivated diphtheria toxin (CRM). Subcutaneous immunization of mice with this conjugate along with complete Freund’s adjuvant (CFA) protected against invasive candidiasis and aspergillosis by 70% and 80%, respectively. A similar conjugation with cholera toxin was equally efficient in curing vaginal candidiasis in Wistar rats (Torosantucci et al., 2005; Pietrella et al., 2010). However, the underlying mechanism by which these conjugates provide protective immune responses is still unknown. Vaccination of Lam-CRM197, a laminarin conjugated with diphtheria toxoid, with MF59 adjuvant significantly reduced the mortality of infected mice (Wang et al., 2015). In another report, β-1,2-mannotriose was conjugated to a peptide fragment from fructose bisphosphate aldolase (Fba) of *C. albicans* and tetanus toxin (TT). Almost all of the mice immunized with β-(Man)3-Fba-TT survived the infection (Xin et al., 2012). Passive transfer studies indicated that antibodies but not the cell-mediated immune responses were responsible for the protection of invasive candidiasis by such vaccinations. In a recent report, KLH-conjugates were generated by covalently linking β-1,2-mannan, N-terminal peptide epitopes of *C. albicans* cell wall phosphomannan complex, and Als1p (rAls1p-N) protein with keyhole limpet hemocyanin (KLH) and human serum albumin (HSA) through homobifunctional disuccinimidyl glutarate (Liao et al., 2019). The study found that the resultant glycopeptides with or without any external adjuvant produced high levels of IgG antibodies in immunized mice, and the obtained antisera cross-reacted with the cell surface of a number of fungi. Although these results advocate the potential use of these conjugates as antifungal vaccines, none of these candidates have been taken to clinical trials.

### Recombinant protein vaccines

Since the live attenuated or killed whole cell vaccines are enriched with antigens and heterogenous in compositions, they may encode many unwanted immunological responses including allergenic and/or reactogenic responses in addition to the desirable ones. Such concerns prompted the researchers to introduce recombinant protein or subunit vaccines as potential vaccine candidates (Petrovsky and Aguilar, 2004). Advancements in genetic engineering, host-pathogen interaction, and cellular immunology have helped vaccinologists to formulate efficient recombinant protein or subunit vaccines. The basic principle involves the transfer and expression of the desired gene encoding an immunogenic antigen related to the virulence and pathogenicity of the fungus to elicit a robust immune response. Here, protein antigens are usually mixed with a suitable adjuvant or a protein carrier, most commonly bacterial toxoids, to develop a robust immune response and stable immunization (Cassone, 2008; Santos and Levitz, 2014). In contrast, live attenuated or killed *Candida* vaccines, recombinant vaccines were usually considered safer as they are free of any infectious agents, stable in the host, and easy to immunize. Several studies were carried out on recombinant vaccines and only the prominent ones have been discussed here. Agglutinin-like sequence (Als) proteins are the cell membrane proteins of *C. albicans* involved in the fungal adherence to host endothelium cells, which ultimately leads to the cause of invasive candidiasis (Hoyer and Cota, 2016). Als1 and Als3 proteins in combination with or without adjuvants have been proposed as vaccine candidates against invasive candidiasis. Subcutaneous immunization of the recombinant N-terminus of Als1 (rAls1p-N) resulted in a survival rate of 50–57 percent subsequent to the lethal challenge of *C. albicans* (Ibrahim et al., 2006). This vaccine was quite effective in both immunocompetent and immunosuppressed mice against both Oropharyngeal candidiasis and *Candida* vaginitis (Spellberg et al., 2005). In the murine model of oropharyngeal and vaginal candidiasis, vaccination with the rAls3-N elicited a robust antibody generation, was more effective and had a higher survival rate than rAls1-N (Spellberg et al., 2006). Notably, it also ensures a safeguard against *S. aureus* infection, implying the carrying of evolutionarily conserved epitopes, shared across such distantly related species (Lin et al., 2009). Furthermore, *Candida* Als3 is structurally similar to a *S. aureus* clumping factor (Spellberg et al., 2008). In a phase I clinical trial, NDV-3A, a rAls3-N vaccine formulated with Alhydrogel adjuvant, increased the antibody titers in revaccinated people at two different doses, including the level of cytokines and IgG and IgA1 titers (Schmidt et al., 2012). Currently, NDV-3A is ongoing in a phase two of clinical trials to assess the vaccine’s immuno-therapeutic efficacy in women with RVVC (Edwards et al., 2018). NDV-3A has also been tested against *C. auris*, and *in vitro* studies revealed that anti-Als3 antibodies developed in the vaccinated mice cross-reacted with this fungus, block biofilm development, and recuperate macrophage-mediated fungal clearance (Singh et al., 2019). Thus, NDV-3A appears to be an extremely promising vaccine candidate for use against C*. albicans* and *C. auris*. Another prominent example in this listing is secretory aspartyl proteases (SAP). SAP is a group of ten secretory proteins of *C. albicans* and they play important roles in fungal cells adhesion, epithelial as well as endothelial invasion, and metabolism (Naglik et al., 2003). Among all, Sap2 is the most abundantly expressed SAP. Intravaginal or intranasal immunization of rats with recombinant Sap2, either with or without cholera toxin as an adjuvant, resulted in *Candida* vaginal infection clearance (De Bernardis et al., 2002). PEV7, a truncated Sap2 protein (77–400 amino acid length) embedded into the bilipid layer of influenza virosomes was also developed by the same research group and has been found to provide efficient protection against vaginal candidiasis (De Bernardis et al., 2012). Intramuscular immunization of PEV7 in mice and rats generate a robust serum antibody response and anti-Sap2 IgG and IgA was also detected in the vaginal fluid of animals. PEV7 has also been advanced to human trials for RVVC treatment. Another study found that recombinant Sap2 protein from *C. parapsilosis* is highly immunogenic and demonstrated that immunization of mice with CpSap2 showed enhanced protection compared to vaccination with Sap2 protein from *C. albicans*, and *C. tropicalis* (Shukla and Rohatgi, 2020). Heat shock protein 90 (HSP-90) is a ubiquitous stress-induced chaperone that plays a critical role in balancing and overcoming the stressful environment within the host cell. It is found in the cell wall of *C. albicans* and is vitally important for yeast survival and viability. The carboxyl terminal 47-kDa fragment of Hsp90 is highly immunogenic, and antibodies against it are associated with a better prognosis, whereas low levels are linked to mortality (Matthews et al., 1987; Matthews and Burnie, 1989; Burnie and Matthews, 2003). Pre-clinical assessment of the efficacy of Mycograb (*Neu*Tec Pharma plc), a human genetically recombinant antibody against heat shock protein 90 (rP-HSP90C) has been carried out recently (Matthews et al., 2003). Mycograb in combination with Amphotericin B provided complete protection for *C. albicans*, *C. krusei*, and *C. glabrata* infections. Another study evaluated the efficacy of chitosan hydrogel (CH-HG) as an adjuvant in recombinant HSP90C protein vaccine (Li et al., 2021). In comparison to free rP-HSP90C, CH-HG-loaded rP-HSP90C produced stable rP-HSP90C-specific IgG, enhanced Th1, Th2, Th17 responses, and a stronger CTL response. Consequently, CH-HG-rP-HSP90C vaccination enhanced protection to pathogenic challenge and increased the survival rate of infected mice. Hyphal-regulated cell wall protein1 (Hyr1) is a GPI-anchored mannon protein present on the fungal cell during hyphal formation. Subcutaneous immunization of a recombinant form of N-terminus of Hyr1 (rHyr1-N) with either CFA or aluminum hydroxide to both immunocompetent and neutropenic mice showed substantial protection to *C. albicans, C. glabrata, C. krusei, C. parapsilosis, and C. tropicalis* infection (Cassone et al., 2010; Luo et al., 2011). This study claimed that the protection is governed by an enhanced antibody titer raised against rHyr1p-N. Consequently, passive immunization with anti-Hyr1p IgG extended the survival of *C. albicans* infected mice. They also suggested the contribution of T- and B-cells in the mechanism of rHyr1p-N governed protection against pathogenesis (Luo et al., 2011).

### Peptide vaccine

Like whole cell vaccines, recombinant proteins also possess several antigenic epitopes, which can result in both the induction of a protective immune response along with adverse and unfavourable ones. Therefore, the concept of peptide vaccines carrying highly desired and specific epitopes was explored (Sesardic, 1993). Peptide vaccines are being considered for preventing and giving protection through active and passive immunization (Xin et al., 2012; Cassone and Casadevall, 2012). Besides, epitope based vaccines are cost effective, less time-consuming, highly efficacious, and safe for use in humans (Backert and Kohlbacher, 2015). Several synthetic vaccine candidates have already been attempted as a therapeutic or prophylactic agent against many diseases like influenza, hepatitis B virus (HBV), hepatitis C virus, HIV, tuberculosis, pneumonia, histoplasmosis, coccidioidomycosis, sporotrichosis, blastomycosis, paracoccidioidomycosis, candidiasis, aspergillosis, cryptococcosis, and other mycoses as well. Active immunization with dendritic cells (DCs) stimulated by either Fba peptide of sequence YGKDVKDLFDYAQE or Met6 peptide of sequence PRIGGQRELKKITE showed protection in both cyclophosphamide-induced neutropenia and healthy mice (Xin, 2016). Further, the protective efficacy of a synthetic Fba peptide (14-mer) conjugated with each one of five peptide mimotopes from Met6 (PS2, PS31, PS28, PS55, and PS76) was also evaluated (Xin et al., 2019). Although all of the five mimotopes elicited specific antibody responses, only three of them protected against invasive candidiasis in mice. Using computational tools, Tarang et al., screened the *Candida* proteome (6030 proteins) and shortlisted specific epitopes belonging to HLA class I, HLA class II, and B-cell. To enhance the vaccine efficacy, a multivalent recombinant protein against *C. albicans* (mvPC) was designed by joining the selected 18-most promising epitopes by molecular linkers. With the addition of a synthetic adjuvant (RS09), it was predicted that with enhanced immunogenicity, mvPC will be a potent vaccine candidate (Tarang et al., 2020). The efficacy of this candidate vaccine against candidiasis is yet to be demonstrated in animal or clinical setups. Several reports suggest that both pathogenic and non-pathogenic bacteria, archaea, and eukaryotic cells secrete extracellular or membrane vesicles (EVs) as a mode for cell-free intercellular communication (Bose et al., 2020). EVs carry a range of cargo compounds that play a role in cellular competition, fitness, survival, invasion, bypassing of the host immune system, establishment, and infection. Especially, fungal species from ascomycetes and basidiomycetes produce EVs that possess a wide range of biologically active molecules that showed a significant degree of virulence (Rodrigues et al., 2011; Freitas et al., 2019; Vargas et al., 2020; Rizzo et al., 2020). For example, when RAW 264.7 macrophages were stimulated with EVs secreted out of *C. albicans* cells produced nitric oxide (NO), IL-12, IL-10, and TGF-β. The bone marrow-derived macrophages (BMDM) upon stimulation by *C. albicans* EVs produced NO, IL-12, tumor necrosis factor alpha (TNF-α), and IL-10, whereas the bone marrow-derived dendritic cells (BMDC) produced IL-12, TNF-α, and TGF-β (Vargas et al., 2020). Despite the promising implications of EVs in vaccine development, like a whole cell vaccine, the structure, and composition of EVs are very complex, thus they may show a wide range of immunological responses, and thereby efficacy of such vaccines remain a concern.

### DNA vaccines

In DNA vaccines, a recombinant plasmid construct containing the cDNA of the desired antigen is transfected into the host’s APCs (mainly DCs) to elicit immunity. In addition to the antigen, the gene encoding the co-stimulatory molecules or cytokines can be inserted into the plasmids as well. For example, a plasmid carrying non-methylated CpGs is sensed by TLR9 (found on DCs), which further induces adaptive immunity. A single study compared the efficacy of two vaccine formulations (recombinant hsp90-CA protein and hsp90-CA-encoding DNA vaccine) to induce protective responses against both systemic and vaginal candidiasis in BALB/c mice. While the intradermal immunization of a DNA vaccine resulted in a 64% prolonged survival duration of mice compared to a PBS control, the intranasal vaccination failed to provide any protection. The intradermal recombinant hsp90-CA protein priming, followed by a booster dose *via* intranasal or intradermal induced a significant increase of hsp90-CA-specific IgG and IgA antibodies in comparison with the control group (Raska et al., 2008).

### Antibody mediated vaccine

Studies to develop an antibody-based diagnosis of various fungal infections are on the rise, and some of the outcomes also look promising. The mAb JF5 for the detection of invasive pulmonary aspergillosis and mAb (CAGTA) for deep-seated *C. albicans* infection are a few of those examples (Thornton, 2008; Martinez-Jimenez et al., 2014). Recently, Rudkin et al. generated seventeen recombinant human anti-*Candida* monoclonal antibodies (mAbs) from single B cells potentially used for diagnosis and therapeutics against pan-fungal infections. They amplified and cloned the human antibody encoding variable domain (V) targeting *C. albicans* epitopes from the cDNA of B cells isolated from recovered patients with mucosal candidiasis. The purified mAbs were found to cross-react with most pathogenic *Candida* species and exhibit strong fungal killing activity *in vitro*, and protect against a lethal challenge in a murine model (Rudkin et al., 2018).

### Pan-fungal vaccine providing cross protection to other pathogens

Infection with a single strain of pathogen occasionally confers protection on a host by preventing infection with a closely relevant strain of that pathogen. In the case of candidiasis, cross protection has also been reported. On a similar line, protection against other fungal or bacterial pathogens by experimental *Candida* vaccines was also found. For example, the subcutaneous immunization with heat-killed *S. cerevisiae* yeasts (HKY) ensured protection against a range of fungal species, including *A. fumigatus, C. albicans*, and *C. posadasii* (Capilla et al., 2009; Liu et al., 2011b). The recombinant Als-3 protein of *C. albicans* also protects against *S. aureus* infection (Lin et al., 2009). It suggests that these distantly related species share common epitopes. Not surprisingly, CaAls3 protein shares structural similarities to a clumping factor found in *S. aureus* (Spellberg et al., 2008). This strategy may be harnessed to generate “convergent immunity” that will protect from diseases caused by pathogens from various kingdoms. Immunization of mice with calnexin in glucan particles showed resistance to a wide range of infections caused by *A. fumigatus, Blastomyces dermatitidis, Fonsecaea pedrosoi, Histoplasma capsulatum*, and *Pseudogymnoascus destructans*, mediated by evoking calnexin-specific CD4+ T cells. In 2015, researchers used genetically modified CD4^+^ T cells to identify an amino acid sequence from the chaperone calnexin protein that showed minimal divergence amongst all Ascomycetes (Wuthrich et al., 2015). Furthermore, a 13-mer peptide (LVVKNPAAHHAIS) derived from the conserved region of calnexin induced a robust immunological response to reduce the severity of *B. dermatitidis* infection (Wuthrich et al., 2015). F-box protein Fbp1 is a *Cryptococcus* virulence factor involved in regulating host-fungus interactions (Wang et al., 2019). The study demonstrated that immunized mice with heat-killed *fbp1Δ* cross-protected fungal pathogens such as *C. neoformans, Cryptococcus gattii*, and *A. fumigatus* by eliciting superior protective Th1 host immunity, albeit less protective against *C. albicans*.

### Nanoparticles as an alternative vaccine

Nanotechnology has already been employed as an alternative to increasing the bio-distribution, treatment effectiveness, and lowering side effects of certain antifungal drugs (Voltan et al., 2016). Antigens can also be delivered by using nanoparticles (NPs). Han and Cutler used liposomes derived from phosphatidylcholine and cholesterol to carry mannan adhesin fraction extracted from *C. albicans* as a potent NPs based *C. albicans* vaccine (Han and Cutler, 1995). Vaccination protected both immuno-competent and -suppressd mice against *C. albicans* and *C. tropicalis*. A specific monoclonal antibody MAb B6.1 was isolated from the immunized mice and was found to protect against widespread infection including RVCC (Han et al., 1998). Another study evaluated *C. albicans* ribosomes trapped in the liposomes of dimyristoyl phosphatidylcholine (DMPC) and dimyristoyl phosphatidyl glycerol (DMPG) as a potential vaccine and found that upon immunization about 60% animals survived with invasive candidiasis (Eckstein et al., 1997). NPs of recombinant HSP90 protein and nickel chelating liposomes associated with norAbuMDP pyrogen adjuvant were injected intradermal to BALB/c mice and a comparable Th1 and Th2 response as in Freund’s complete adjuvant vaccine was observed (Masek et al., 2011). Similarly, Knotigová et al. evaluated rHSP90 in nickel chelating liposomes associated with two pyrogen-free adjuvants (norAbuMDP and norAbuGMDPs) as a potential NP-based vaccine in ICR mice and rabbits (Knotigova et al., 2015). Recently monoolein based liposomes for delivery of *C. albicans* cell wall proteins Cht3p and Xog1p were explored (Carneiro et al., 2015). In another study, ADS1 and ADS2 formulations were evaluated which differed only in lipid concentrations for cell wall protein loading, found that ADS1 but not ADS2 protected against fungal infection in mice (Carneiro et al., 2016).

## Vaccines in various stages of clinical trials

Although several candidate vaccines have been identified, and they appear to be efficient and safe in animal models, only two candidates have reached clinical trials Phase I in humans. The first vaccine uses alum as an adjuvant and the N-terminus of a recombinant Als3 protein of *C. albicans* as antigen (Nova Digm, US). NDV-3A is the first vaccine to demonstrate preclinical efficacy in protecting from diseases caused by both fungal and bacterial pathogens (novadigm.net). According to a phase I clinical trial, the NDV vaccine was found to be nontoxic and effectively produced antibody and T-cell immune responses in healthy individuals (Schmidt et al., 2012). Seventy three adult volunteers were immunized with two doses of NDV-3A. After the 1^st^ dose of immunization, all the individuals produced anti-Als3p IgG antibodies in comparison to placebo as a control. After the second dose, a robust IgA1 antibody titer with effective IgG response, and IL-17 and IFN-γ T cells cytokines production were observed in all the subjects. The phase 2 randomized, double-blind, placebo-controlled clinical trial has also been recently completed, and the report suggested that one-dose of NDV-3A vaccine was also safe and effective in patients with recurrent vulvovaginal candidiasis (RVVC). The vaccine reduced the frequency of vulvovaginal candidiasis for up to 12 months in women under 40 years old (Edwards et al., 2018). However, NDV-3A vaccination in a population of military trainees did not impede the nasal or oral acquisition of *S. aureus* (Millar et al., 2021). Another vaccine being conducted in the clinical trial (Phase I) was on the Sap2 protein of *C. albicans* in virosomal formulation against RVVC (PEV7, Pevion Biotech AG, Switzerland) (Sandini et al., 2011; De Bernardis et al., 2012). PEV7 was safe and effective, and the vaccinated individuals produced specific and functional B cell memory (www.pevion.com). We expect that these clinical studies will be further extended to larger cohorts including immunosuppressed individuals or at least to individuals receiving corticosteroids and antibiotics in the future.

## Challenges in developing a successful anti-candidiasis vaccine and future perspective

Among the estimated ~4 million fungal species on the planet Earth, about 300 species are pathogenic and cause diseases in humans (Stop neglecting fungi, 2017; Hawksworth and Lucking, 2017). While most fungal infections are found in immune-deficient individuals, some fungi cause diseases even in healthy adults. In addition to these diversified fungal pathogens, the site of fungal infections also varies widely, from the scalp of the head to the nails of the toes. Although these infections are superficial and not life threatening, occasionally the current regime of treatment using fungal drugs becomes ineffective. Moreover, systemic fungal infections are very serious as they target most of the internal organs through the circulatory system in animals and humans. Additionally, some fungi do not have terrestrial life rather they survive as commensals in humans and animals and might regulate host physiology. Most of the studies so far considered *C. albicans* only as a pathogenic yeast causing both primary and secondary infections, however being a commensal, it may maintain a mutualistic relationship with the host that has not been explored yet. Our recent study suggests a mutualism between *C. albicans* and mice, and *C. albicans* modulating gut microbiota, metabolism, and immunity for the benefit of the host (Peroumal et al., 2022). In that context, using anti-fungal drugs will only cause dysbiosis similar to antibiotics and will enhance the severity of fungal and other secondary infections. At the same time, this long association of fungi and the host also suggests that both humans and *C. albicans* have evolved to recognize and develop some kinds of escape mechanisms to protect each other. The fact that the immunocompetent host is rarely affected by *Candida* infections again implements that humans have developed immunotolerance towards the commensal fungal pathogens. In contrast, to escape the host immune defense systems, *C. albicans* adapts and evades by altering its shape, size, and genetic makeup. Morphological and genome plasticity is frequently found in clinical isolates of *C. albicans*. Probably, the immune memory of the host fails to recognize this newly evolved pathogen in immune compromised situations. Secondly, immune compromised patients may not respond to vaccines. Therefore, conceptually, it becomes difficult and challenging to develop drugs and immunotherapeutics against *Candida* species. While designing a suitable drug or a vaccine, it is important to take into account that it should not target the commensal state of *C. albicans* or other such fungi as it may be deleterious to the host development, and those should be equally effective in immune suppressed individuals. One way to overcome this issue is to validate drug and vaccine candidates at the preclinical stage itself by using humanized and immunodeficient mice models such as SCID or Nude rather than using inbreed animals. Further, they need to be revalidated using higher animals. It is most likely that the antigenic-peptide based vaccines may not differentiate between commensal and pathogenic states of *Candida*, in that context, a whole cell vaccine specifically designed against the pathogenic form has the advantage. Among the *Candida* species, *C. albicans* is the frequently clinically isolated pathogen. However, reports suggest that *C. parapsilosis* is frequently found in children, whereas *C. glabrata* is more prevalent among older aged adults. Thus, the candidate vaccines should also target *C. albicans* as well as non-albicans species. Since birth, *C. albicans* and non-albicans species evolve with humans. Ironically, although their life cycle operates in humans, we are not immune to fungal infections. They are highly likely to adopt to various niches in the human gut and other sites to evade the host’s defense system. In fact, genetic, phenotypic, and morphological plasticity is commonly found in *C. albicans* and other non-albicans species. Therefore, fungal infections today have become one of the most challenging diseases to manage in humans. In addition to the vaccine, the adjuvant also plays an important role in the additional activation of T- or B-cells, thereby enhancing the immune response. The antigenicity of the immunogen is also greatly enhanced by the presence of a suitable adjuvant. Freund’s adjuvant and alum adjuvant are commonly used in animals and humans, respectively. In the future, in order to achieve better efficacy without side effects, new adjuvants or modified adjuvants should be tested. Since fungal infections those are invasive and occur in immuno-compromised individuals, it will be challenging to have a vaccine that is equally effective in healthy and weaker individuals. Normally, the vaccine elicits either weak or no immune response in immune deficient individuals, in such a scenario, passive immunotherapy could be the other option. Passive immunotherapy, also known as adoptive immunotherapy, requires direct administration of immune system components, such as monoclonal antibodies, activated macrophages, etc. As discussed earlier, monoclonal antibody C7 (MAb C7) and Mycograb are some of the examples used in animals, however, their use, safety, and effectiveness for human use are yet to be determined.

Early diagnosis, identification of novel antifungals with high safety and low side effects, prophylactics, and immune-therapeutics are the need of the hour to combat fungal infections. Undoubtedly, we need a safe and effective fungal vaccine (s), however, challenges are immense both conceptually as well as on technical fronts to develop a successful vaccine against Candidiasis and other mycoses. So, the design of an effective vaccine should be such that (i) it should be highly immunogenic, (ii) it should protect against a wide range of fungal pathogens, (iii) it will not only target the market appealing superficial but also it should protect from bloodstream infections, and (iv) more importantly, it will be equally effective in individuals with compromised immunity. Thus, an approach to developing a pan-fungal vaccine should be ideal. In our laboratory, we have generated an array of DNA polymerase subunit knockouts of *C. albicans*, and some of them exhibit reduced or constitutive filamentation. A few of those strains show altered cell wall architectures and slow growth phenotypes (Acharya et al., 2016; Peroumal et al., 2019). Their ability to develop systemic candidiasis and protection against pathogenic challenges is being explored to develop a whole-cell vaccine. Such knockouts may be generated for non-albicans species as well as to develop multivalent vaccine strains. Although there is a long way to go to develop a successful *Candida* vaccine, the future looks promising, and with the efforts of several immunologists working in the field, it will be feasible to have a multivalent vaccine similar to as against viruses and bacteria.

## Conclusion

Despite those fungal diseases are equally critical and fatal as viral and bacterial diseases, the nonavailability of broad-spectrum antifungals, early diagnostics, and approved vaccines clearly indicate the need for rigorous and coordinated efforts from researchers, public health authorities, and funding agencies. Global estimates suggest about a billion people getting affected by fungal infections and over 1.5 million infected people get killed by fungal diseases every year. Fungal diseases are still not taken seriously by health care providers probably as the fungal infections occur mostly as secondary to a primary disease such as AIDS, cancer, organ transplantation, etc. Therefore, a delay in treatment results in serious illness, organ dysfunction, and death. In the last two pandemic years, we have witnessed a rise in fungal infections due to SARS-CoV2 infections, and many of those infected patients succumbed to various mold infections. Since vaccination has been the major preventive measure for several infectious diseases, a safe and effective multivalent vaccine that targets the *Candida* species is a strong medical need of the hour to avoid deaths. More importantly, the vaccine should target both systemic candidiasis and mucosal infections. As the whole cell vaccines against viruses and bacteria are widely accepted, a similar approach may be undertaken to identify stable and more antigenic live attenuated strains of *C. albicans* that may protect a wide range of fungal infections. Considering the importance of trained immunity, such vaccines may also provide cross protection against bacterial infections.

## Author contributions

NA conceptualized and designed the manuscript, SS, SB, MS, PK, AD, BU, and SKP prepared an initial draft of the manuscript. NA wrote the final draft. NA obtained the funding. All authors contributed to the article and approved the submitted version.

## Acknowledgments

We thank our other laboratory colleagues for their inputs and helpful discussions. Financial support to NA’s laboratory by Institutional core grant, DBT (BT/PR15470/MED/29/997/2015 and BT/PR32817/MED/29/1495/2020), and SERB (EMR-2016-000640) are greatly acknowledged.

## Conflict of interest

The authors declare that the research was conducted in the absence of any commercial or financial relationships that could be construed as a potential conflict of interest.

## Publisher’s note

All claims expressed in this article are solely those of the authors and do not necessarily represent those of their affiliated organizations, or those of the publisher, the editors and the reviewers. Any product that may be evaluated in this article, or claim that may be made by its manufacturer, is not guaranteed or endorsed by the publisher.

## Abbreviations

CDC, Centers for Disease Control and Prevention; DOX, doxycycline; NK, natural killer; DC, dendritic cells; HKY, heat killed yeast; GXM, glucuronoxylomannan; HSA, human serum albumin; GUTIs, genitourinary tract infections; TT, tetanus toxoid; CFA, complete Freund’s adjuvant; Als, Agglutinin-like sequence; NP, nanoparticle.
